# Texture Analysis in Volumetric Imaging for Dentomaxillofacial Radiology: Transforming Diagnostic Approaches and Future Directions

**DOI:** 10.3390/jimaging10110263

**Published:** 2024-10-22

**Authors:** Elaine Dinardi Barioni, Sérgio Lúcio Pereira de Castro Lopes, Pedro Ribeiro Silvestre, Clarissa Lin Yasuda, Andre Luiz Ferreira Costa

**Affiliations:** 1 Postgraduate Program in Dentistry, Cruzeiro do Sul University (UNICSUL), São Paulo 1506-000, SP, Brazil; elainedinardi2@gmail.com; 2 Department of Diagnosis and Surgery, São José dos Campos School of Dentistry, São Paulo State University (UNESP), São José dos Campos 2245-000, SP, Brazil; sergioluciolopes@gmail.com (S.L.P.d.C.L.); pedro.silvestre@unesp.br (P.R.S.); 3 Laboratory of Neuroimaging, Department of Neurology, University of Campinas (UNICAMP), Campinas 13083-970, SP, Brazil; cyasuda@unicamp.br

**Keywords:** computer-assisted diagnosis, cone-beam computed tomography, feature extraction, magnetic resonance imaging, radiomics

## Abstract

This narrative review explores texture analysis as a valuable technique in dentomaxillofacial diagnosis, providing an advanced method for quantification and characterization of different image modalities. The traditional imaging techniques rely primarily on visual assessment, which may overlook subtle variations in tissue structure. In contrast, texture analysis uses sophisticated algorithms to extract quantitative information from imaging data, thus offering deeper insights into the spatial distribution and relationships of pixel intensities. This process identifies unique “texture signatures”, serving as markers for accurately characterizing tissue changes or pathological processes. The synergy between texture analysis and radiomics allows radiologists to transcend traditional size-based or semantic descriptors, offering a comprehensive understanding of imaging data. This method enhances diagnostic accuracy, particularly for the assessment of oral and maxillofacial pathologies. The integration of texture analysis with radiomics expands the potential for precise tissue characterization by moving beyond the limitations of human eye evaluations. This article reviews the current trends and methodologies in texture analysis within the field of dentomaxillofacial imaging, highlights its practical applications, and discusses future directions for research and dental clinical practice.

## 1. Introduction

Texture analysis has gained prominence in dentomaxillofacial imaging modalities by offering new perspectives on how oral and medical radiologists interpret and use imaging data. This advanced technique provides additional methods to assess radiographic images, thus complementing the traditional visual assessments. By leveraging sophisticated image processing algorithms, texture analysis enables the non-invasive extraction of quantitative information from medical images [1], which enhances diagnostic accuracy and treatment planning in oral and maxillofacial pathology [2,3].

In the realm of medical and dental imaging, texture analysis serves as a quantitative method to evaluate the distribution and relationship of pixel or voxel intensities within a specified region of interest (ROI) or volume of interest (VOI) [4,5]. For instance, a rough-textured image has a high rate of change in pixel intensity compared to a smooth-textured one. This technique allows for detailed characterization of images by providing valuable insights into tissue structures and pathologies which may not be apparent through traditional visual inspection [1,6].

By analyzing the patterns of gray-level transitions in medical images, a unique set of textural characteristics can be identified, often described as a “texture signature.” This texture signature may serve as a distinctive marker for accurately characterizing specific pathological processes [7].

Radiomics is a technique in the field of medical imaging that focuses on extracting a large number of quantitative features from medical images [8,9]. These features, known as “radiomic features”, can include measures of texture, shape, intensity, and more, and are used to create detailed profiles of tissue characteristics [9,10]. Radiomics goes beyond traditional visual analysis by enabling the identification of complex and subtle patterns that may be associated with different pathological states [5,11,12]. By integrating this information with clinical and genomic data, radiomics has the potential to significantly enhance diagnostic accuracy, treatment personalization, and therapeutic response monitoring across various medical fields, including oral and maxillofacial conditions [8,11,13].

The relationship between texture analysis and radiomics is synergistic. Although radiomics encompasses a broader range of quantitative features, texture analysis focuses specifically on spatial relationships and patterns within the image data [4,12]. Together, they enable radiologists to go beyond size-based or human eye-dependent semantic descriptors, offering a more comprehensive understanding of imaging data [4,5,11].

The application of texture analysis in head and neck imaging has seen significant advancements in recent years, particularly the widespread adoption of cone-beam computed tomography (CBCT) scanners. This narrative review explores the current landscape of texture analysis in dentomaxillofacial radiology by examining its practical applications in various oral and maxillofacial conditions, including evaluation of oral mucosa. Furthermore, this article delves into cutting-edge methodologies of texture analysis, discusses standardization efforts for quality assurance, and offers insights into future directions promising to further enhance tissue characterization and treatment monitoring in the field of oral radiology.

## 2. Search Strategy and Methodology

To ensure a comprehensive and up-to-date review of texture analysis and radiomics in dentomaxillofacial imaging, we conducted a systematic literature search across multiple databases. It serves as a basis for discussing current trends and potential advancements in the field. The following electronic databases were queried: Google Scholar; PubMed; Scopus; and Web of Science.

Our search strategy employed a combination of relevant keywords to capture the breadth of research in this field. The primary search terms included the following:

- “texture analysis AND (dentomaxillofacial OR oral OR maxillofacial)”;
- “radiomics AND (dentomaxillofacial OR oral OR maxillofacial)”;
- “(CT OR MRI OR CBCT) AND texture analysis AND dentomaxillofacial”.

Additionally, we conducted a supplementary search to identify key articles defining and explaining the concepts of texture analysis and radiomics in medical imaging. This supplementary search used terms such as the following:

- “texture analysis definition AND medical imaging”;
- “radiomics principles”;
- “medical image feature extraction fundamentals”.

These foundational articles were included regardless of their specific application area, as they provide essential context and methodological background for the review.

To capture a wide range of the relevant literature, we also used variations and combinations of these terms. The search was limited to English-language articles published in peer-reviewed journals. We focused on the literature from the past five years to ensure the review reflects the most current advancements in the field, although seminal papers from earlier years were also included when deemed essential to the understanding of fundamental concepts.

### 2.1. Inclusion and Exclusion Criteria

Inclusion criteria:

- Studies focusing on texture analysis or radiomics in dentomaxillofacial imaging;
- Original research articles, systematic reviews, and meta-analyses;
- Articles exploring texture analysis applications in related fields with potential relevance to dentomaxillofacial imaging;
- Studies published in English.

Exclusion criteria:

- Case reports and conference abstracts;
- Articles without full-text availability;
- Studies using non-sectional imaging modalities (e.g., panoramic radiographs);
- Articles lacking substantial discussion or application of texture analysis techniques in medical imaging contexts.

### 2.2. Study Selection Process

The study selection process involved the following steps:

(a) Initial screening of titles and abstracts to identify potentially relevant articles;
(b) Full-text review of selected articles to determine eligibility based on the inclusion and exclusion criteria;
(c) Reference list screening of included articles to identify additional relevant studies.

Two authors (E.D.B. and A.L.F.C.) independently performed the selection process, with any disagreements resolved through discussion or consultation with a third author (S.L.P.d.C.L).

Our initial search yielded a total of 16,747 potentially relevant articles across the databases:

Google Scholar: 15,364 articles;

PubMed, Scopus, and Web of Science combined: 1383 articles.

These articles underwent a screening process based on our inclusion and exclusion criteria. After thorough evaluation of titles, abstracts, and full texts where necessary, we selected 57 articles for inclusion in the final review. The majority of these studies were in vivo human studies, focusing on clinical applications of texture analysis. We also included a limited number of in vitro studies that provided valuable methodological insights or validation of techniques applicable to clinical settings. No animal studies were included in this review.

### 2.3. Data Extraction and Synthesis

For each included study, we extracted key information such as study design, sample size, imaging modalities used, texture analysis or radiomic features examined, and main findings. The extracted data were synthesized narratively, focusing on emerging trends, methodological advancements, and clinical applications in dentomaxillofacial radiology.

## 3. Historical Perspective of Texture Analysis in Radiology

### 3.1. Texture Analysis Methods

Texture analysis in radiology has its roots in the fundamental concept of image texture, which refers to the spatial variation in pixel intensity levels within an image [14]. This technique emerged as a mathematical method to evaluate the grayscale and its relationships with adjacent ones, helping to characterize the tissue under study [6].

In its early stages, texture analysis included various models to assess image characteristics accurately. These models can be broadly categorized into three main types: statistical-based, model-based, and transform-based methods [6,15]. Among these, the statistical-based method has become the most common approach in texture analysis [6].

Statistical methods of texture analysis are further divided into different orders of measure parameters as follows [1,16,17]:

(a) First-order statistics: it analyzes the frequency distribution in the region of interest through histogram. First-order statistics do not consider pixels around the ROI and measure parameters such as intensity, standard deviation, skewness, and kurtosis;
(b) Second-order statistics: it uses a gray-level co-occurrence matrix (GLCM) to explore how often pairs of pre-determined pixel values occur within a spatial range in the image, representing the joint probability density function of intensity levels occurring in a certain direction at specified distances;
(c) Higher-order statistics: it examines the overall differences between pixels or voxels within the context of the entire region of interest. Higher-order statistics often use neighborhood gray-tone-difference matrices to obtain metrics such as variations within the image and the spatial rate of gray-level change.

### 3.2. Evolution of Imaging Modalities

As imaging technologies advanced, so did the application of texture analysis in radiology. The technique has been successfully applied to various imaging modalities, including computed tomography (CT) and magnetic resonance imaging (MRI) [1,6,14].

CT and MRI cross-sectional imaging techniques provided a non-invasive method to identify and characterize tumors by using texture analysis [7,18,19]. The application of texture analysis to CT and MRI in oncology represents a significant advancement in quantitative imaging, offering a more comprehensive assessment of tumors beyond the traditional visual interpretation [20]. As research in this field continues to grow, texture analysis is increasingly being integrated into clinical decision-making processes, enhancing the role of radiology in personalized cancer care [21].

The advancement of imaging modalities has allowed texture analysis to be applied to several medical fields. For instance, it has been used to evaluate changes in ischemic stroke, osteoarthritis, and osteoporosis [22,23,24]. In the realm of internal medicine, texture analysis has been used to better characterize hepatic fibrosis, emphysema, and liver cirrhosis, although this research is still in the experimental phase [6,25].

## 4. Milestones in Dentomaxillofacial Applications

**Figure 1:**
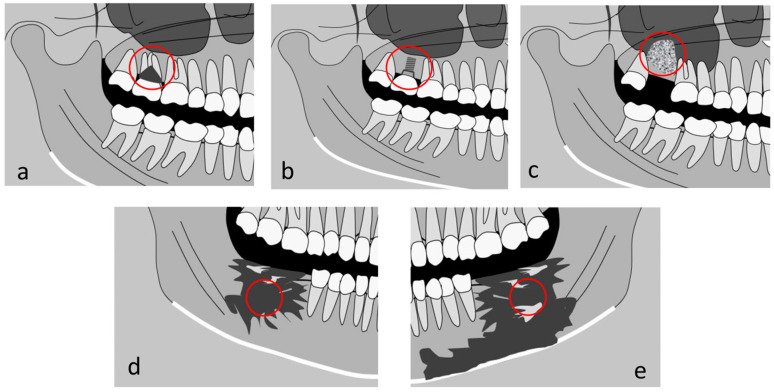
Examples of application of texture analysis to dentomaxillofacial images: (**a**) periodontal application to dentomaxillofacial images; (**b**) predictive study on the stability of dental implants; (**c**) study on sinus-lift grafts; (**d**) characterization of malign lesions; and (**e**) study on osseous lesions due to medication-related necrosis.

In dentistry, texture analysis has made significant strides, particularly in the field of oral radiology. Some key milestones in its application include the following (Figure 1):

- In periodontal health assessment: Goncalves et al. [26] demonstrated a significant advancement in furcal lesion detection using texture analysis (TA) of CBCT images. Their study revealed statistically significant differences (*p* < 0.05) in almost all texture parameters when comparing lesion areas (with intermediate areas and control areas);
- In evaluation of the stability of dental implants: Costa et al. [27] investigated the use of texture analysis on CBCT images to evaluate dental implant stability. Their study found significant correlations between texture parameters and implant insertion torque. Higher contrast in the peri-implant bone was associated with higher insertion torque (*p* < 0.001), while higher entropy in the implant bone site (position S1.0) correlated with lower torque (*p* = 0.006). These findings suggest that texture analysis of CBCT images could potentially predict implant stability, offering valuable insights for treatment planning in dental implantology;
- In bone graft evaluation: Azimzadeh et al. [28] studied texture analysis of CBCT images following sinus lift surgery using allograft and xenograft materials. The study involved 42 patients and analyzed 11 texture parameters. Results showed no significant differences in primary outcomes between xenograft and allograft groups. However, the allograft group displayed statistically higher average gray-level values;
- In oral cancer assessment: de Oliveira et al. [29] conducted a study on texture analysis of multi-slice spiral computed tomography images in head and neck squamous cell carcinoma (HNSCC) with 46 patients. The study analyzed eleven GLCM parameters to assess tumor differentiation grades and showed significant correlations between texture parameters and histopathological grades of tumor differentiation. The findings suggest that texture analysis could serve as an age-independent biomarker for HNSCC;
- In bone analysis in the medication-related osteonecrosis of the jaw (MRONJ): Queiroz et al. [30] analyzed CBCT images of 16 MRONJ patients using texture analysis. They found significant differences (*p* < 0.05) in texture parameters among active osteonecrosis, intermediate tissue, and healthy tissue areas. Intermediate and active osteonecrosis areas showed higher values in contrast, entropy, and secondary angular momentum compared to healthy tissue, indicating greater tissue disorder. This suggests texture analysis could improve accuracy in determining MRONJ extent, potentially aiding treatment planning.

One of the significant advantages of texture analysis in dentomaxillofacial applications is its ability to provide detailed information about bone involvement in border regions between pathologically affected areas and healthy ones. This has been particularly useful in cases of MRONJ as texture analysis has revealed that visually unaffected areas may show altered behavior due to osteonecrosis [30] or even underscoring the sensitivity in identifying subtle changes in mandibular bone marrow that precede clinical symptoms, potentially allowing for earlier intervention and improved patient outcomes [31].

As the field continues to evolve, researchers are exploring the potential of texture analysis for early diagnosis of various oral and maxillofacial conditions. This application is already being used by neurologists for the early diagnosis of ischemic stroke and for quantifying the real extent of the affected area [32].

Recent studies have further expanded the applications of texture analysis in dental imaging. Bayat et al. [33] conducted a randomized clinical trial evaluating radiographic texture analysis in the context of socket preservation using allograft and xenograft materials. Their findings highlighted significant changes in hard tissue texture following dental implantation procedures, emphasizing the potential of texture analysis in monitoring post-operative bone healing and integration.

In another study, Muraoka et al. [34] explored the use of MRI texture analysis for the quantitative evaluation of acute osteomyelitis in the mandibular bone. This research demonstrated that texture analysis could effectively quantify inflammatory changes, offering a non-invasive tool for early diagnosis and management of osteomyelitis [2].

Moreover, Muraoka et al. [35] assessed the diagnostic efficacy of combining apparent diffusion coefficient (ADC) values with texture features in differentiating odontogenic cysts and tumors. This approach enhanced diagnostic accuracy, suggesting that texture analysis, when combined with other imaging metrics, can significantly improve the differential diagnosis of complex maxillofacial lesions.

De Rosa et al. [3] and Yomtako et al. [36] both explored the differentiation of periapical granulomas from radicular cysts using texture analysis, albeit with different CT modalities. De Rosa et al. [3] utilized CBCT to analyze texture features, demonstrating its effectiveness in distinguishing these lesions based on their textural characteristics. Similarly, Yomtako et al. [36] applied texture analysis to multi-CT images, achieving comparable differentiation between radicular cysts and granulomas.

These studies collectively demonstrate the expanding role of texture analysis in dental radiology, providing valuable insights into tissue characterization and enhancing diagnostic precision across various conditions and imaging modalities.

## 5. Image Acquisition Protocols for Texture Analysis

Image acquisition plays a key role in the radiomics workflow, particularly regarding the texture analysis. The consistency of acquisition and reconstruction protocols is essential for obtaining reliable and reproducible results, especially in multicenter studies [14]. This section explores the key considerations for different imaging modalities used in dentomaxillofacial radiology.

### 5.1. CT Imaging Parameters for Texture Analysis

CT has been widely used for texture analysis in various medical applications. However, variations in acquisition and reconstruction parameters can lead to inconsistent findings between different datasets [14]. Phantom studies have shown that interscanner and intrascanner differences in radiomic metrics can be significant. For instance, Mackin et al. [37] demonstrated that the variability in radiomic metrics extracted from CT images of a phantom was comparable to that observed in non-small-cell lung carcinoma tumors.

To ensure the quality and repeatability of radiomic studies using CT, it is essential to maintain consistency in image acquisition and reconstruction protocols. This includes standardizing parameters such as slice thickness, field of view (FOV), and reconstruction algorithms across different scanners and institutions.

### 5.2. MRI Sequences for Texture Analysis

MRI offers excellent soft-tissue contrast and the ability to enhance different types of tissues using various acquisition protocols [38]. However, the choice of MRI sequence for texture analysis depends on the specific application and the tissues being studied [39].

MRI-based texture analysis involves several key considerations that significantly impact the quality and reliability of results. Sequence selection is decisive, as different MRI sequences produce varying texture patterns. For instance, contrast-enhanced T1-weighted images are often preferred for brain tumor assessment, although diffusion-weighted images have shown efficacy in tumor classification. Image resolution, determined by slice thickness, FOV, and matrix size, plays a vital role in texture analysis sensitivity. Higher spatial resolution images tend to be more susceptible to variations in acquisition parameters. The signal-to-noise ratio (SNR) is another critical factor, with higher levels improving texture discrimination. Scanners with higher field strength, such as 3T, provide an increased SNR, potentially leading to enhanced texture-based discrimination. Preprocessing steps are essential for optimal results, with ROI normalization and correction of non-uniformity artifacts being recommended procedures. Finally, the quantization of gray-levels is fundamental for texture analysis methods based on matrix computation. These considerations, as outlined in the literature, form the foundation for effective MRI-based texture analysis, ensuring that the resulting data are both accurate and clinically relevant [38].

### 5.3. CBCT Parameters for Texture Analysis

CBCT has become a valuable tool in dentomaxillofacial radiology, offering detailed three-dimensional (3D) images of oral and maxillofacial structures. When employing CBCT for texture analysis, several critical factors must be considered to ensure reliable and accurate results.

Image quality is a primary concern in CBCT-based texture analysis: understanding texture analysis techniques and their applications in radiology is decisive for effective implementation [14]. Despite the potential for increased noise and lower image quality compared to conventional CT, research has demonstrated that select radiomic metrics show robustness in CBCT images when consistent imaging protocols are used. Studies have revealed that radiomic features can be reproducibly measured from CBCT images, underscoring the importance of standardized imaging protocols in mitigating the impact of noise and enhancing the reliability of texture metrics [40].

Motion artifacts present another challenge in CBCT imaging: research has addressed this issue, finding that limited breathing-related motion has a reasonable impact on radiomic metrics [40]. This highlights the need to account for patient movement during image acquisition to maintain the integrity of radiomic data. Consideration of motion artifacts is necessary for ensuring the reproducibility and reliability of texture analysis results.

The selection of the ROI and the segmentation process are vital steps in ensuring accurate texture analysis: recent initiatives in image biomarker standardization stress the importance of standardized quantitative radiomics for high-throughput image-based phenotyping [41]. This research emphasizes the need for meticulous attention to detail and consistency in ROI selection and segmentation to produce reliable results across different studies and patient populations.

Software considerations play a significant role in CBCT texture analysis. Specialized software can be employed to analyze CBCT images effectively. The choice of software is particularly important given the unique challenges posed by CBCT data, including noise and resolution differences. Studies have demonstrated the reproducibility of radiomic features, highlighting the necessity of using reliable software tools that can adequately handle these specific challenges [40].

## 6. Image Segmentation

Image segmentation, a critical first step in texture analysis and radiomics, involves delineating the ROI in two-dimensional (2D) or VOI in 3D approaches. This process defines the specific area from which radiomic features will be extracted and calculated [4,10].

Image segmentation in radiomics can be approached through three main methods, each with its own advantages and limitations. Manual segmentation, often considered the gold standard, involves experts meticulously delineating regions of interest. Although this method provides high-quality results, it is time-consuming and susceptible to both intra- and inter-observer variability. Studies employing manual segmentation should assess the reproducibility of derived radiomic features and exclude those that prove non-reproducible from further analyses. Semi-automatic segmentation offers a middle ground, utilizing standard image segmentation algorithms such as region-growing or thresholding, followed by manual corrections. This approach is faster than fully manual segmentation but still introduces some level of observer bias. Automatic segmentation, a rapidly evolving field, employs advanced techniques including deep learning algorithms, often utilizing a U-Net architecture. This method’s primary advantage lies in its ability to eliminate intra- and inter-observer variability. However, a significant challenge remains in ensuring the generalizability of trained algorithms across diverse datasets. Each of these approaches plays a decisive role in radiomics research, with the choice depending on the specific requirements of the study, available resources, and the need for reproducibility and efficiency [4,10].

Various software solutions, both open-source and commercial, are available for image segmentation including the following [10]:

- 3D Slicer (https://www.slicer.org/);
- ImageJ (https://imagej.net/);
- Invesalius (https://invesalius.github.io/);
- LifEx (https://www.lifexsoft.org/);
- MeVisLab (https://www.mevislab.de/de/);
- MITK (https://helmholtz.software/software/mitk);
- ITK-SNAP (http://www.itksnap.org/pmwiki/pmwiki.php);
- OsiriX (https://www.osirix-viewer.com/).

The choice of segmentation method significantly impacts the results of a radiomics study. Although automatic or semi-automatic segmentation improves reproducibility, it may be less accurate due to artifacts and noise. Deep learning techniques and neural networks have found extensive application in automated segmentation, as well as in feature extraction and selection [4,9].

Importantly, the decision on what to segment (e.g., 3D image, 2D image, multiple levels on a single slice, ROI, and VOI) can lead to variability in study outcomes. Semi-automatic techniques involving automatic segmentation with secondary reading by a radiologist can improve reproducibility but may be more time-consuming than manual segmentation [4,42].

As research progresses, efforts are being directed towards developing robust and generalizable algorithms for automated segmentation to overcome current limitations and enhance the reliability of texture analysis methodologies [4].

## 7. Texture Analysis: Feature Extraction and Selection Methods

In the earlier sections of this article, we introduced the main approaches to texture analysis in dental and medical imaging. This section aims to provide a more comprehensive and in-depth exploration of the methodologies used for feature extraction and selection. As previously mentioned, texture analysis uses statistical methods to examine and describe the relationships between gray-level values in an image by typically using commercial software or custom-built tools. Here, we will delve deeper into the three primary approaches: first-order statistical texture analysis, second-order statistical texture analysis, and higher-order statistical texture analysis.

### 7.1. First-Order Statistical Texture Analysis

Bulleted first-order statistical texture analysis focuses on the statistical properties of individual pixel values, without considering their spatial relationships. This method computes various statistics from the histogram of pixel intensities in the ROI [1]. Common first-order features include the following [17]:

- Mean: average intensity value;
- Variance: measure of the intensity distribution;
- Skewness: asymmetry of the intensity distribution;
- Kurtosis: peakedness of the intensity distribution;
- Energy: sum of squared elements in the histogram;
- Entropy: measure of the randomness in pixel intensities.

These features provide a basic characterization of the texture but do not capture spatial relationships between pixels.

### 7.2. Second-Order Statistical Texture Analysis

All second-order statistical texture analysis considers the spatial relationships between pairs of pixels [14]. The most common approach in this category is based on the GLCM, as described by Haralick et al. [43].

GLCM uses second-order statistics to assess the gray level distribution of pairs of pixels in the ROI. Each element (i, j) of this matrix shows how many times gray level i co-occurs with gray level j for a given distance d (usually d = 1, 2, 3, 4, or 5 pixels) and direction *θ* (*θ* = 0°, 45°, 90°, or 135°) [3].

From the GLCM, several texture descriptors can be computed. These features provide more detailed information about texture patterns and are often more discriminative than first-order statistics, including the following [3]:

- Contrast: measure of local variations;
- Correlation: linear dependency of gray levels on neighboring pixels;
- Energy: sum of squared elements in the GLCM;
- Homogeneity: closeness of the distribution of elements to GLCM diagonal;
- Entropy: measure of randomness in pixel pair distributions.

### 7.3. Higher-Order Statistical Texture Analysis

Higher-order statistical texture analysis methods consider relationships between three or more pixels. These techniques can capture more complex texture patterns not detectable with first- or second-order methods [14]. However, they may also be more computationally intensive and result in a larger number of features [1].

Higher-order statistical and transform-based methods offer advanced approaches to texture analysis in dentomaxillofacial imaging. The Gray Level Run Length Matrix (GLRLM) analyzes pixels with the same gray level in a specific direction, effectively capturing coarse textures. Local Binary Patterns (LBPs) consider the relationship between a central pixel and its neighbors, creating a binary code that describes local texture patterns. Gabor filters provide multi-resolution representation of texture features by analyzing textures at different scales and orientations. Similarly, wavelet transforms decompose the image into different frequency bands, enabling multi-scale texture analysis. Fractal-based approaches analyze the self-similarity of textures across different scales, which can be particularly useful for natural textures such as those found in bone structures. These methods, while computationally more complex, offer deeper insights into texture characteristics, potentially revealing subtle tissue changes not visible to the human eye or captured with simpler methods [1,14].

### 7.4. Strengths and Limitations of Texture Analysis Techniques

The comparison of texture analysis methods reveals a spectrum of approaches, each with its own strengths and limitations. First-order statistical methods offer simplicity and computational efficiency, providing a basic characterization of overall image intensity distribution [1,4]. Although useful for initial assessment of tissue homogeneity, they are limited in capturing spatial relationships between pixels and may miss subtle texture patterns important for detecting early-stage pathologies [1,4]. In contrast, second-order statistical methods, particularly those based on GLCM, capture spatial relationships between pixel pairs, making them more sensitive to subtle texture changes and effective in characterizing tissue heterogeneity [1,3]. However, these methods are more computationally intensive and sensitive to image noise and artifacts [1,4].

Higher-order statistical methods excel at capturing complex texture patterns beyond pixel pairs, detecting subtle tissue changes not visible to the human eye [1,2]. They are particularly useful for analyzing heterogeneous structures but come with the highest computational complexity and may produce a large number of features requiring careful selection [5,6]. Model-based methods, such as fractal analysis, offer unique advantages in capturing scale-invariant texture properties, proving useful for analyzing structures with self-similarity like trabecular bone [23,24]. Although less sensitive to image noise, they may oversimplify complex biological structures and have limitations in capturing directional texture information [23,25].

Transform-based methods, exemplified by the wavelet transform, provide the ability to analyze textures at multiple scales, capturing both spatial and frequency information [7,14]. They are effective in detecting localized texture changes but can be challenging in terms of choosing the appropriate wavelet basis and interpreting results clinically [7,14,15]. Each method finds its niche in dentomaxillofacial imaging applications. First-order methods are suitable for quick assessment of bone density variations, although second-order methods excel in differentiating periapical lesions and assessing trabecular bone structure [3,26]. Higher-order methods prove valuable in detailed analysis of bone microarchitecture for implant planning and advanced characterization of TMJ disorders [2,29]. Model-based methods are particularly useful in analyzing bone quality for dental implant placement and evaluating craniofacial growth patterns [27,28]. Transform-based methods find applications in detecting subtle enamel defects and analyzing periodontal ligament space [30,31].

The choice of texture analysis method in dentomaxillofacial imaging ultimately depends on the specific clinical application, image characteristics, and available computational resources [8,9,10]. A combination of methods often yields optimal results, with first-order methods used for initial screening followed by more advanced techniques for detailed analysis of regions of interest [11,12,13]. The integration of multiple texture analysis approaches, along with clinical context and other imaging biomarkers, significantly enhances diagnostic accuracy and treatment planning in dentomaxillofacial radiology [12,13].

### 7.5. Approaches to Feature Selection and Extraction

Feature selection and extraction techniques are often used after texture analysis to reduce dimensionality and select the most relevant features for a given task. Common approaches include principal component analysis (PCA), linear discriminant analysis (LDA), and various machine-learning-based feature selection methods [44]. By combining these different approaches to texture analysis and applying appropriate feature selection techniques, researchers can develop robust and effective methods for characterizing and analyzing textures in various applications, such as medical imaging, remote sensing, and material science [45].

## 8. Interpretation of Extracted Features

Haralick et al. [43] proposed a comprehensive set of texture descriptors which are foundational in the field of image analysis, particularly in the context of GLCM-based methods. These descriptors are designed to capture various texture features in an image, thus providing insights into the spatial relationships and intensity variations among pixel pairs so that information about underlying tissue structures, pathologies, or anatomical variations can be obtained. These texture features are described below:

(a) Contrast: this feature measures the intensity variation between pixels. High contrast values often indicate heterogeneity within a ROI, which may correspond to pathological conditions. For instance, increased contrast has been observed in malignant lesions, such as squamous-cell carcinoma, where the tissue heterogeneity is more pronounced due to irregular cell arrangements;
(b) Inverse difference moment: this feature assesses the uniformity of pixel pairs. A higher homogeneity value suggests that the ROI has similar pixel intensities, which could be indicative of benign conditions or healthy tissue. For example, benign odontogenic tumors may exhibit higher homogeneity compared to malignant tumors due to their more uniform tissue structure;
(c) Angular second moment: this feature represents the uniformity of the texture and is often associated with smooth textures. Higher energy values can indicate more regular or homogenous structures, as seen in healthy bone or dental tissues, where the pixel intensities are more consistent across the ROI;
(d) Correlation: this feature measures the linear dependency of gray levels on those of neighboring pixels. A high correlation may reflect organized structures, such as the layered organization seen in dental enamel or the regular patterns in compact bone. In contrast, lower correlation might be associated with disorganized tissue structures, such as those seen in inflammatory conditions;
(e) Sum of squares: variance measures the distribution of gray-levels within the ROI. A higher variance might suggest a more complex texture, which could correlate with pathological changes. For instance, higher variance has been reported in cases of periodontitis where the bone structure becomes irregular due to disease progression;
(f) Entropy: entropy measures the randomness in the texture. Higher entropy values suggest a more complex and disordered texture, which may be seen in malignant or inflamed tissues where the cellular architecture is disrupted. For example, lesions with high cellular atypia or necrotic areas, such as those found in aggressive tumors, often present higher entropy;
(g) Sum average, sum variance, and sum Entropy: these features further analyze the distribution of pixel values. Higher values of sum entropy, for example, are associated with greater disorder within the tissue, which can be seen in advanced stages of malignancies. On the other hand, sum variance might increase in cases where the texture becomes more heterogeneous, as observed in the progression of dental caries;
(h) Difference of variance and difference of entropy: these features capture the variations and entropy differences within the ROI. Significant differences may indicate transitions between different tissue types or the presence of pathological processes altering the tissue architecture, such as in fibrous dysplasia or cystic lesions.

These examples highlight how specific GLCM features can be linked to pathological conditions, enhancing the diagnostic capability of dentomaxillofacial radiology. By integrating these features into diagnostic workflows, clinicians can better differentiate between healthy and pathological tissues, improving treatment outcomes.

**Figure 2:**
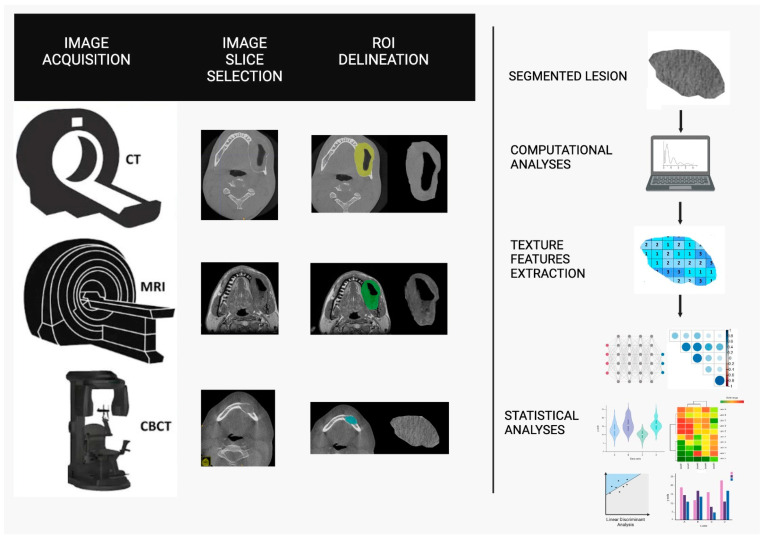
Overview of the texture analysis process: starting from scanner acquisition, followed by slice selection, ROI and segmentation of a lesion of the jaws, leading to extracted features, and concluding with statistical analysis (created with BioRender.com).

Figure 2 summarizes the steps of the texture analysis process in images.

## 9. Practical Application of Texture Analysis in Advanced Dentomaxillofacial Imaging

Texture analysis has become an innovation in dentomaxillofacial radiology, with the potential to greatly improve diagnostic precision and enhance patient outcomes. However, interpreting texture analysis can be challenging due to the complexity of the data and the need for specialized expertise. Despite these challenges, its incorporation into imaging reports can provide invaluable insights that improve clinical decision-making.

One of the primary applications of texture analysis in dentomaxillofacial imaging is in the assessment of bone quality and changes. For instance, studies have demonstrated its utility in evaluating hard tissue changes post socket preservation, providing detailed insights that are fundamental for dental implant planning [33]. This capability allows for a more precise assessment of bone healing and quality, which can lead to improved surgical outcomes and patient satisfaction.

In the context of temporomandibular joint (TMJ) disorders and other maxillofacial pathologies, texture analysis offers a non-invasive method to detect subtle changes in bone and soft tissue. This method is particularly valuable in assessing the TMJ disc and surrounding structures, where traditional imaging may not fully capture the complexity of pathological changes.

Recent studies have highlighted the utility of texture analysis in TMJ disorders. For instance, Girondi et al. [46] demonstrated that texture analysis of MRI images can effectively identify disc changes associated with effusion in the TMJ. This approach provides detailed insights into the structural alterations of the joint, which are decisive for accurate diagnosis and management of TMJ disorders [1]. Similarly, Luo et al. [47]. explored the application of MRI-based texture analysis in evaluating the lateral pterygoid muscle in young patients with temporomandibular disorders. Their study revealed that texture analysis could detect fasciculation and other subtle muscle changes, which are often indicative of underlying dysfunction. These findings underscore the potential of texture analysis to enhance the understanding of muscle involvement in TMJ disorders, facilitating more targeted therapeutic interventions.

Furthermore, texture analysis has shown promise in the monitoring and evaluation of periodontal disease and other chronic conditions. By providing quantitative data on bone density and structural changes, it supports clinicians in tracking disease progression and response to treatment over time. This ongoing assessment is necessary for managing chronic conditions effectively and preventing complications.

In the context of salivary gland imaging, texture analysis offers a formidable tool for enhancing diagnostic precision. Nardi et al. [48] showed that MR diffusion-weighted imaging combined with texture analysis can effectively differentiate parotid gland lesions, providing critical insights beyond conventional imaging. Similarly, Jiang et al. [49] utilized CT-based texture analysis to distinguish between benign and malignant salivary gland lesions, aiding in early and accurate diagnosis. Additionally, Ito et al. [50] showed that texture analysis could assess parotid sialadenitis, enabling better monitoring of inflammatory changes.

As highlighted in the medical radiology literature, there is a need for standardized protocols in image acquisition and processing to ensure the reliability of textural features [51].

## 10. Critical Evaluation

Texture analysis in dentomaxillofacial radiology stands at a critical juncture, poised between promising advancements and significant challenges. This section critically evaluates the current landscape, dissecting the strengths, weaknesses, and significant gaps in existing research.

### 10.1. Limitations and Challenges

Radiomics faces several significant challenges that impact its widespread adoption and reliability in clinical settings. These challenges can be categorized into three main areas: lack of standardization, complex data interpretation, and limited large-scale validation.

Complex data interpretation is another substantial challenge. Lambin et al. [51] described the extraction of over 200 quantitative features from medical images, including intensity, shape, and texture characteristics. This high-dimensional nature of texture features makes interpretation challenging, especially for clinicians not specialized in advanced image analysis.

Regarding the lack of standardization, Kumar et al. [52] highlight significant variations in imaging parameters. Their study of 74 patients revealed slice thickness variations from 1 mm to 5 mm and pixel size differences from 0.59 mm to 0.98 mm. These inconsistencies affect the information extracted with image feature algorithms, which in turn impacts classifier performance.

Gillies et al. [8] discussed the challenges in quantitative imaging, particularly in PET-CT. They emphasize that quantitative imaging requires not only calibration of the scanner and standardization of the scan protocol but also strict adherence to patient protocols. This further underscores the complexity of standardization in radiomics.

The limited availability of large-scale, multi-center validation studies hinders the generalizability of radiomics findings. Aerts et al. [12] emphasize the importance of external validation in their study on gene-expression analysis for survival prediction in lung cancer. They stress that without proper validation, the risk of overfitting increases, potentially leading to overoptimistic results.

Parmar et al. [42] conducted a study comparing various feature selection and classification methods in radiomics. They found that the choice of classification method is the most dominant source of performance variation, highlighting the need for standardized approaches in radiomics analysis.

### 10.2. Research Gaps and Opportunities for Advancement

More research is needed to assess the value of texture analysis in monitoring disease progression and treatment response over time. Current studies primarily focus on single time point imaging, limiting the understanding of how radiomic features evolve throughout the course of treatment [53]. The concept of delta-radiomics, which involves extracting quantitative features from image sets acquired at multiple time points during treatment, shows promise in improving diagnosis, prognosis, and assessment of therapeutic response [54].

The integration of radiomics results with clinical and histopathological data for comprehensive patient assessment remains limited [8]. Although some studies have explored the combination of radiomic features with genomic data, there is a need for more extensive research integrating radiomics with other clinical parameters. This integration could lead to more robust and clinically relevant prediction models, potentially enhancing personalized treatment strategies [8].

A significant challenge in radiomics is the standardization of imaging protocols and feature extraction methods. This standardization is necessary to ensure reproducibility and comparability across studies. Efforts should be made to develop large, multi-institutional databases to facilitate external validation of radiomic models and improve their generalizability [55].

The integration of artificial intelligence, particularly deep learning algorithms, with texture analysis holds promise for more robust and automated analysis [56]. Nurzynska et al. [57] conducted a study comparing texture analysis with deep learning approaches for differentiating age and sex in vertebral body CT scans. Their research demonstrates the potential of integrating advanced machine learning techniques with radiomics to enhance the analysis of medical images, particularly in the context of bone structure evaluation.

## 11. Conclusions and Outlook

The integration of texture analysis into dentomaxillofacial radiology marks a significant stride towards personalized dental care. This review highlights key insights and outlines strategic directions for advancing research and clinical applications in this field. The development of quantitative imaging biomarkers through texture analysis shows great promise, necessitating validation through large-scale, multi-center clinical trials to establish their reliability and clinical utility. This detailed image analysis enables more individualized treatment planning, and future efforts should focus on integrating these results into decision-making algorithms and treatment protocols to optimize therapeutic interventions.

However, the widespread adoption of texture analysis faces challenges, primarily the lack of standardized protocols in image acquisition and processing. To address this, we recommend establishing international working groups to develop and implement standardized guidelines. Bridging the gap between research and clinical practice requires the development of user-friendly software tools that seamlessly integrate texture analysis into existing radiological workflows, alongside educational programs to train dentomaxillofacial radiologists in effectively interpreting and utilizing texture analysis data.

A fundamental aspect of clinical integration is the incorporation of texture analysis interpretations into imaging reports. General practitioner dentists, who may lack specialized training in advanced image analysis techniques, often find it challenging to interpret raw texture data. By including expert interpretations of texture analysis results in standard imaging reports, radiologists can provide general dentists with understandings derived from these advanced techniques. This approach ensures that the valuable information obtained through texture analysis is effectively communicated and utilized in clinical decision-making, bridging the gap between advanced imaging technology and practical patient care.

In conclusion, although texture analysis in dentomaxillofacial radiology faces challenges in standardization and clinical integration, its potential to enhance diagnostic capabilities and support personalized patient care is significant. By addressing these challenges through focused research, education, and interdisciplinary collaboration, the field can move towards realizing the full benefits of texture analysis in routine clinical practice. This progression will not only improve the quality of dental and maxillofacial healthcare, but also pave the way for more precise, personalized, and effective patient management strategies in the future.

## Data Availability

Not applicable.
